# Recurrent Infections After Percutaneous Pinning of a Proximal Radius and Ulna Fracture

**DOI:** 10.5435/JAAOSGlobal-D-23-00081

**Published:** 2023-10-06

**Authors:** Federico Galar, Clinton Ulmer, Steven Gibbons, Sekinat McCormick, Matthew Landrum

**Affiliations:** From the Department of Orthopedic Surgery, UT Health San Antonio, San Antonio, TX (Mr. Galar, Dr. Ulmer, Dr. Gibbons, Dr. McCormick, and Dr. Landrum); Orthopaedic Surgery, University of Arkansas Medical Sciences, Little Rock, AR (Dr. Landrum).

## Abstract

Elbow fractures in the pediatric population are an exceedingly common injury, comprising 5% to 10% of all pediatric fractures, with supracondylar fractures being the most common of the subset. Radial neck fractures are less frequent, comprising only 1% of all pediatric fractures. We provide a case report of a 7-year-old girl with a left radial neck and proximal ulna fracture. A 7-year-old girl presented to the emergency department after falling off a rock wall the day before. Imaging showed a left proximal ulna and proximal radial neck fracture. The patient underwent percutaneous reduction and fixation, complicated by subsequent infection requiring surgical débridement. The patient then developed a recurrent infection 1 year later, requiring repeat irrigation and débridement. The patient has since made a full recovery, returned to activities of daily living, and regained a full range of motion. Radial neck fractures have a low incidence and have been frequently associated with poor outcomes. The main mechanism by which these fractures occur is due to falling on an outstretched arm. Percutaneous pinning is often recommended after unsuccessful attempts at closed reduction because open reduction is often complicated by postoperative stiffness. As with any procedure that involves breaking the skin, there is a risk of infection. However, there is unclear evidence regarding ideal perioperative management to prevent postoperative infection.

Proximal radius fractures^1,2,3,4^ comprise 5% to 10% of all pediatric elbow fractures,^5^ with rates of osteomyelitis localized to the radius and ulna having been reported as low as 0.005% and 0.014%,^6^ respectively. Osteomyelitis has proven to increase the risk of treatment failure among pediatric fractures. Furthermore, recurrence of infection has been seen in less than 10% of treated osteomyelitis.^6^ Limited data have been reported regarding the symptomatology at the onset of proximal radius fracture postoperative infection. However, Schroeder et al^7^ reported diagnosis of supracondylar fracture infections at an average of 25 days postoperatively with all infections. All these patients received standard preoperative antibiotic prophylaxis. Concern regarding the validity of administration of antibiotics perioperatively has arisen given the concern for increased resistance of pathogens and lack of consistent evidence demonstrating effective reduction of infection among the pediatric orthopaedic population.

The purpose of this case report was to describe the treatment course of a patient with a rare injury and an even rarer complication. This includes both lessons learned and questions raised during the care process.

## Case Report

The patient is a 7-year-old girl who was admitted to the emergency department after falling off a rock wall from a height of 6 feet the day before. She was evaluated at an outside hospital and diagnosed with a forearm fracture. The patient was found to have tenderness to palpation over the elbow and difficulty performing motor examination secondary to pain. Sensation was grossly intact.

## Imaging

**Figure 1:**
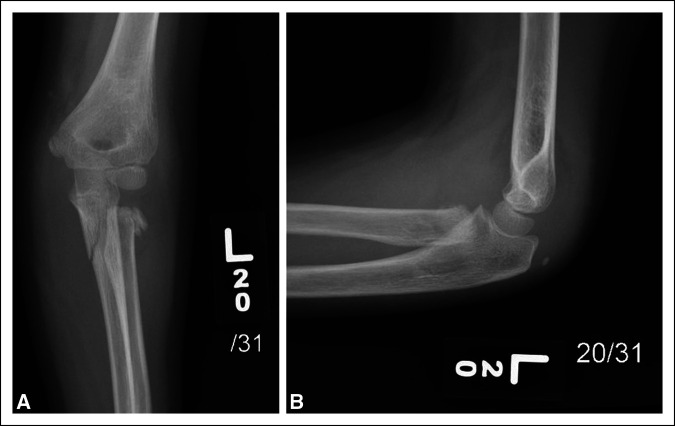
Radiographs of AP and lateral views of the left elbow showing proximal ulna and proximal radius with radial neck fracture.

Radiographs of the left elbow and forearm demonstrated a minimally displaced fracture of the left proximal ulna and Salter Harris type 2 fracture of the proximal radius involving the radial neck with total displacement of the proximal fracture fragment (Figure 1).

## Surgery

**Figure 2:**
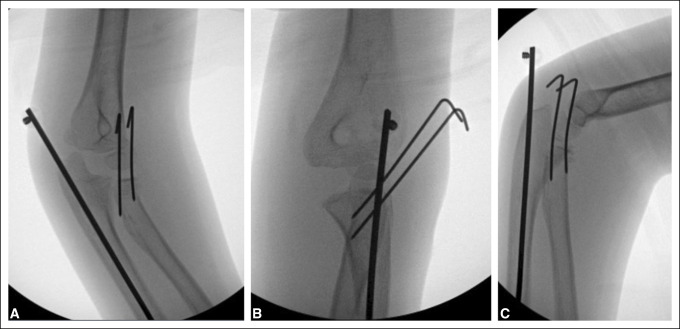
Radiographs of intraoperative fluoroscopy showing reduction and fixation of fractures.

The patient was taken to the operating theater the next day. She was positioned supine. Two attempts at closed reduction were made using Israeli flexion-distraction direct pressure and the Patterson technique with an Esmarch tourniquet. Both attempts were unsuccessful. A stab incision over the proximal dorsal forearm was then made, and blunt dissection performed. Using fluoroscopic guidance and traction, a percutaneous Kirschner wire reduction was performed using the blunt end of a 3/32 Kirschner wire to reduce the radial head. Two percutaneous 0.054 Kirschner wires were placed to ensure stability and prevent redislocation of the radial head. The forearm was kept in pronation to avoid injury to the posterior interosseus nerve during this process. After correction of the radial fracture, a stab incision was made over the olecranon, and a 2.4-mm Steinmann pin was introduced and driven intramedullary across the fracture site into the intramedullary canal of the ulna to prevent displacement of the ulna and radial head. Both Kirschner wires and the Steinmann pin were cut and a Jergens ball placed to prevent migration beneath the skin. Fluoroscopy confirmed reduction and assured stability on AP and lateral views (Figure 2). Stab incision sites were irrigated copiously and closed. The patient was then placed in a long arm cast.

## Postoperative Course

**Figure 3:**
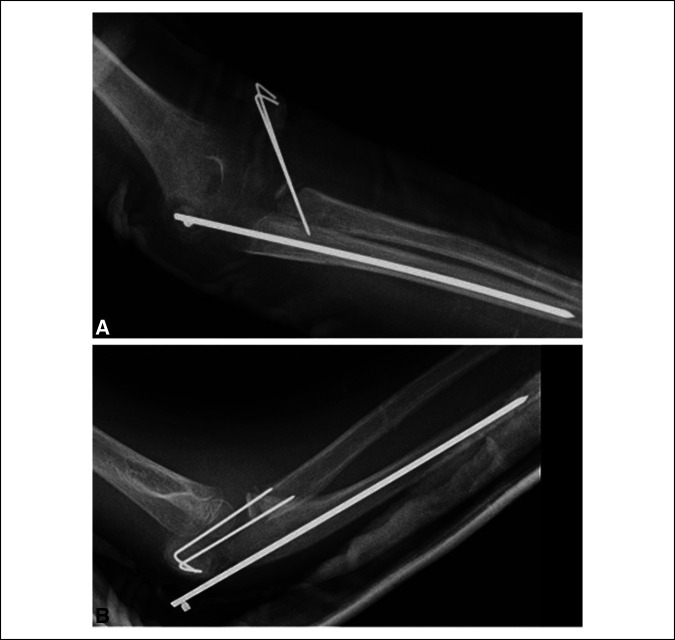
Radiographs showing two views of the left elbow before pin removal.

The patient was discharged the same day of her surgery. Two and a half weeks later, the patient presented to the emergency department with worsening fevers and increasing left elbow pain. The patient was administered a one-time dose of ceftriaxone 1,320 mg in 0.9% sodium chloride. The cast was removed, and purulence was noted on cast padding. No drainage was expressed from the pin sites. At the time, imaging demonstrated no evidence of implant complication or cortical erosions, and the patient was placed in a posterior slab splint (Figure 3). The patient was placed on cephalexin 250 mg/5 mL orally four times daily for 10 days. The patient had pins removed 4 days later in the outpatient setting.

**Figure 4:**
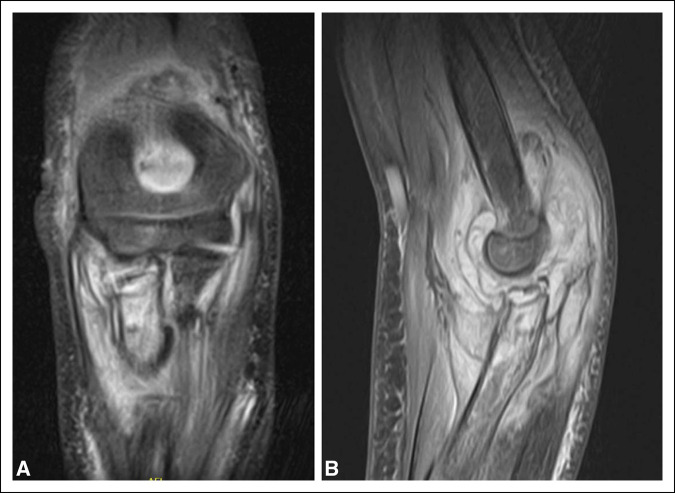
Radiographs showing T2 coronal and sagittal MRI slices concerning for elbow septic arthritis and osteomyelitis in the proximal ulna.

Three weeks later, plain radiographs demonstrated effusion suggesting concern for infection. An MRI was ordered in the setting of elevated erythrocyte sedimentation rate and C-reactive protein, which demonstrated a notable joint effusion with concern for septic arthritis and osteomyelitis of the proximal radius (Figure 4). The patient was taken to the operating theater for left elbow arthrotomy and drainage. A sinus tract and granulation tissue were appreciated from the lateral pins where the radial pinning had been performed. The granulation tissue was excised, and dissection was done to identify the extensor digitorum communis, which was then split. The intermuscular septum superficial to the distal humerus was identified and split to facilitate the capsulotomy as well. Within the elbow joint, frank purulence was noted, was collected, and sent for culture. Inflamed synovial tissue was appreciated and sent for culture. A 2.0 drill bit was then used to place two drill holes unicortically through the proximal radius and three holes into the distal humerus, which released a small amount of purulence. The olecranon fossa was noted to be negative for purulence. The elbow joint and subsequent drill holes were thoroughly irrigated, and a small Penrose drain was placed in the elbow joint. The incisions were closed, and the left upper extremity was placed in a soft dressing. Cultures returned negative for growth. The patient was continued on the aforementioned Keflex regimen for additional 3 weeks. One month postoperatively, the patient was assessed and noted to have full extension of left elbow and lacked approximately 20° to 30° of flexion of the same elbow. In addition, she lacked approximately 5° of full supination in comparison with the contralateral side and had full pronation. No signs of erythema or drainage at the incision site were appreciated. The patient was given home exercise programs for rehabilitation.

**Figure 5:**
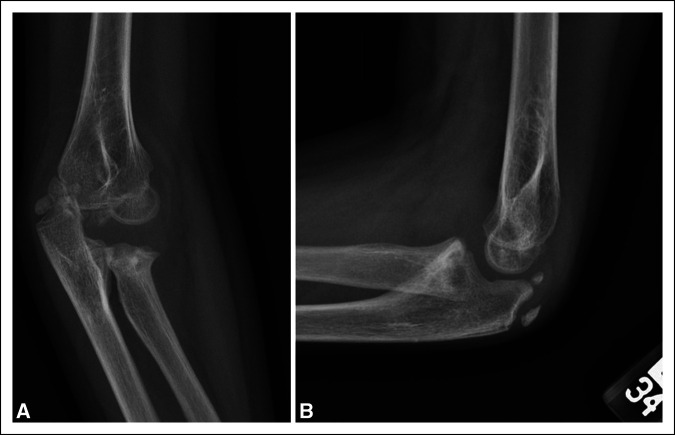
Radiographs of AP and lateral views of the left elbow showing moderate effusion at 1-year mark.

**Figure 6:**
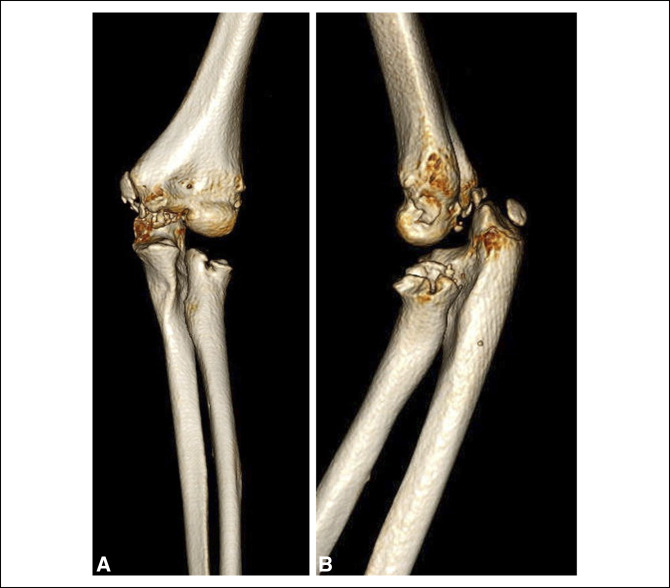
Illustrations showing CT 3D reconstructions of left elbow osteolysis at the 1-year mark.

**Figure 7:**
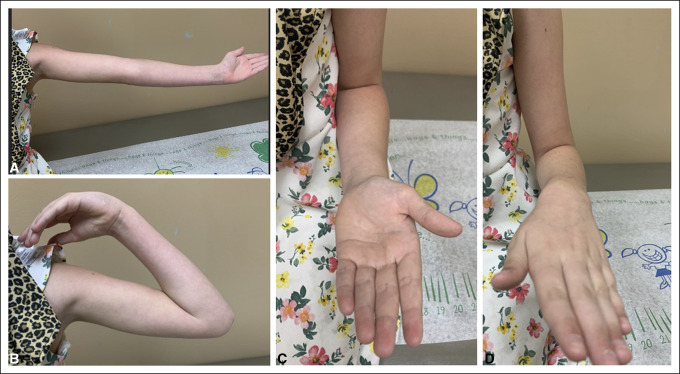
Clinical photographs showing full range of motion at the elbow 3 months after repeat incision and drainage.

One year after initial fixation, the patient presented to the emergency department for progressive left elbow swelling and pain after falling on her arm 2 weeks earlier. Imaging demonstrated elbow effusion with chronic destructive changes of the radial head and olecranon (Figures 5 and 6). The patient underwent subsequent irrigation and débridement where purulence was noted within the capsule. In addition, a bone biopsy of the proximal radius core was performed using a Jamshidi needle. Additional irrigation was performed, and the skin was closed. A sterile dressing was placed. Cultures and bone biopsy returned negative for growth. The patient was placed on clindamycin, per the recommendation of the infectious disease physician, for a duration of approximately 5 weeks. At the 3-month follow-up, imaging demonstrated continued remodeling of the proximal radius and ulna without the evidence of displacement or dislocation. The patient denied any additional pain and was able to return to her activities of daily living with full range of motion (Figure 7).

## Discussion

Proximal radial fractures with involvement of the neck remain an uncommon finding in pediatric orthopaedics. The management of these fractures depends on the angle of displacement with recommendations of attempting closed reduction in those fractures with angle of displacement greater than 30°. If closed reduction is unsuccessful, percutaneous reduction should be attempted before open reduction.^8^ Closed reduction options include push and lever techniques with the use of intramedullary nails or, as in the case of this patient, Kirschner wires and Steinmann pins when a fracture fragment is deemed unstable with necessary follow-up for surgical removal of these wires and pins.^9^

Furthermore, infection and delayed recurrent infection are a rare complication sparse in the current literature. As in this case, delayed pin tract drainage and septic arthritis can be a presenting sign of osteomyelitis. Identification of these features on imaging with the usage of MRI is paramount to the timely management of osteomyelitis, with nearly 40% of these individuals requiring additional surgical interventions. Untreated osteomyelitis increases the risk of permanent cartilage damage and growth arrest, as is seen in other osteoarticular infections. As such, cultures, irrigation and débridement, and proper antibiotic coverage are required in the proper management of these cases with close follow-up even after antibiotic cessation.^6,10-12^

Surgical management is essential in the acute setting. The goals of surgery are to decompress and irrigate the joint, fenestrate osteomyelitic bone, and obtain tissue samples for tailored antibiotics. Once a diagnosis of septic arthritis is obtained, surgical débridement should be expeditious. As in this case, it is essential to consider intra-articular bony structures that may have osteomyelitis because purulence that breaks through and intra-articular metaphyseal cortex will seed a joint with bacteria.

Controversy regarding the prophylactic administration of antibiotics has surfaced. Unnecessary prophylactic administration may be leading to an increase in resistance among pathogens. Bloomer et al and Bashyal et al reported a lack of statistical significance in infection rates among those who were treated prophylactically with antibiotics and those who went untreated in the setting of proper preoperative sterilization techniques in pediatric supracondylar fractures.^13,14^ However, other studies analyzing comprehensive antibiotic management to include prophylaxis, such as that by Vanderberg et al, have found statistical significance in the development of surgical site infections in those who were noncompliant with the guidelines laid out by their comprehensive management.^15^ These incongruous results may be driving the prophylactic administration of antibiotics by pediatric orthopaedic surgeons and the subsequent potential development of resistant organisms without clear guidelines and statistical data.^16^

In conclusion, proximal radial neck fractures remain a rare incidence in the pediatric population, particularly with the recurrence of osteomyelitis. Antibiotic prophylaxis of these individuals may not necessarily change their intended clinical course, as is being seen with the recently published literature. Regardless, with proper postoperative management, patients can go on to have promising recovery and return to their activities of daily living without long-term sequalae.

## References

[1] Hart ES Turner A Albright M Grottkau BE: Common pediatric elbow fractures. Orthop Nurs 2011;30:11-19. 21278549 10.1097/NOR.0b013e31820574c6

[2] Cossio A Cazzaniga C Gridavilla G Gallone D Zatti G: Paediatric radial neck fractures: One-step percutaneous reduction and fixation. Injury 2014;45(suppl 6):S80-S84. 25457324 10.1016/j.injury.2014.10.028

[3] Zimmerman RM Kalish LA Hresko MT Waters PM Bae DS: Surgical management of pediatric radial neck fractures. J Bone Joint Surg Am 2013;95:1825-1832. 24132355 10.2106/JBJS.L.01130

[4] Nicholson LT Skaggs DL: Proximal radius fractures in children. J Am Acad Orthop Surg 2019;27:e876-e886. 30865025 10.5435/JAAOS-D-18-00204

[5] Kang S Park SS: Predisposing effect of elbow alignment on the elbow fracture type in children. J Orthop Trauma 2015;29:e253-e258. 25756916 10.1097/BOT.0000000000000322

[6] Yi J Wood JB Creech CB : Clinical epidemiology and outcomes of pediatric musculoskeletal infections. J Pediatr 2021;234:236-244.e2. 33771580 10.1016/j.jpeds.2021.03.028 PMC8238832

[7] Schroeder NO Seeley MA Hariharan A Farley FA Caird MS Li Y: Utility of postoperative antibiotics after percutaneous pinning of pediatric supracondylar humerus fractures. J Pediatr Orthop 2017;37:363-367. 26558958 10.1097/BPO.0000000000000685

[8] Radomisli TE Rosen AL: Controversies regarding radial neck fractures in children. Clin Orthop Relat Res 1998:30-39. 10.1097/00003086-199808000-00005 9728157

[9] Shlykov MA Milbrandt TA Abzug JM Baldwin KD Hosseinzadeh P: Displaced radial neck fractures: What are my options? Instr Course Lect 2019;68:375-382. 32032046

[10] Jardaly AH LaCoste K Gilbert SR Conklin MJ: Late deep infections complicating percutaneous pinning of supracondylar humerus fractures. Case Rep Orthop 2021;2021:7915516. 34631185 10.1155/2021/7915516 PMC8497162

[11] Browne LP Mason EO Kaplan SL Cassady CI Krishnamurthy R Guillerman RP: Optimal imaging strategy for community-acquired Staphylococcus aureus musculoskeletal infections in children. Pediatr Radiol 2008;38:841-847. 18560822 10.1007/s00247-008-0888-8

[12] Griswold BG Sheppard E Pitts C Gilbert SR Khoury JG: The introduction of a preoperative MRI protocol significantly reduces unplanned return to the operating room in the treatment of pediatric osteoarticular infections. J Pediatr Orthop 2020;40:97-102. 31923170 10.1097/BPO.0000000000001113

[13] Bloomer AK Coe KM Brandt AM Roomian T Brighton B Scannell BP: Hold the antibiotics: Are preoperative antibiotics unnecessary in the treatment of pediatric supracondylar humerus fractures? J Pediatr Orthop 2022;42:e474-e479. 35200212 10.1097/BPO.0000000000002118

[14] Bashyal RK Chu JY Schoenecker PL Dobbs MB Luhmann SJ Gordon JE: Complications after pinning of supracondylar distal humerus fractures. J Pediatr Orthop 2009;29:704-708. 20104149 10.1097/BPO.0b013e3181b768ac

[15] Vandenberg C Niswander C Carry P : Compliance with a comprehensive antibiotic protocol improves infection incidence in pediatric spine surgery. J Pediatr Orthop 2018;38:287-292. 27280896 10.1097/BPO.0000000000000812 PMC5145789

[16] Malone SM Seigel NS Newland JG Saito JM McKay VR: Understanding antibiotic prophylaxis prescribing in pediatric surgical specialties. Infect Control Hosp Epidemiol 2020;41:666-671. 32252848 10.1017/ice.2020.71 PMC8202117

